# Clinical and Obstetric Aspects of Pregnant Women with COVID-19: A Systematic Review

**DOI:** 10.1055/s-0041-1733913

**Published:** 2021-12-21

**Authors:** Sarah Nilkece Mesquita Araújo Nogueira Bastos, Bárbara Louise Freire Barbosa, Larisse Giselle Barbosa Cruz, Rayza Pereira de Souza, Simone Santos e Silva Melo, Caroline Camargo Bandeira da Silveira Luz

**Affiliations:** 1 Departamento de Medicina, Universidade Federal do Delta do Parnaíba, Parnaíba, PI, Brazil; 2 Departamento de Medicina, Faculdade Uninassau, Parnaíba, PI, Brazil

**Keywords:** pregnancy, coronavirus infections, severe acute respiratory syndrome, SARS virus, betacoronavirus, gravidez, infecções por coronavírus, síndrome respiratória aguda grave, vírus da SARS, betacoronavírus

## Abstract

**Objective** To analyze the clinical and obstetric aspects of pregnant women with COVID-19.

**Methods** A systematic literature review in the *MEDLINE/PubMed* , *LILACS* , SCIELO, and CNKI databases was performed from March to May 2020, with the descriptors: *Pregnancy* ; *2019-nCov* ; *Coronavirus* ; *SARS-Cov-2* , *Covid-19* . Of those chosen were original titles, without language and period restriction and that addressed pregnant women with a clinical and/or laboratory diagnosis of COVID-19. Revisions, editorials, and duplicate titles were excluded. The Newcastle-Ottawa (NOS) and Murad et al. scales were used to assess the quality of the studies.

**Results** We included 34 articles with 412 pregnant women infected with severe acute respiratory syndrome (SARS-Cov-2), with an average age of 27.5 years of age and 36.0 gestational weeks. The most common symptom was fever (205 [49.7%]), and 89 (21.6%) pregnant women progressed to severe viral pneumonia. Laboratory tests showed an increase in C-reactive protein (154 [37.8%]), and radiological tests showed pneumonia with peripheral ground-glass pattern (172 [51.4%]). Emergency cesarean delivery was indicated for most pregnant women, and the most common gestational complication was premature rupture of ovarian membranes (14 [3.4%;]). We detected 2 (0.5%) neonatal deaths, 2 (0.5%) stillbirths, and 1 (0.2%) maternal death.

**Conclusion** Pregnant women with COVID-19 presented a clinical picture similar to that of non-infected pregnant women, with few obstetric or neonatal repercussions. There was a greater indication of cesarean deliveries before the disease aggravated, and there was no evidence of vertical transmission of the infection.

## Resumo

**Objetivo** Analisar os aspectos clínicos e obstétricos de gestantes com COVID-19.

**Métodos** Revisão sistemática da literatura nas bases: *MEDLINE/PubMed* , *LILACS* , SCIELO e CNKI, realizada de março a maio de 2020, com os descritores *Pregnancy* ; *2019-nCov* ; *Coronavirus* ; *SARS-Cov-2* , *Covid-19* . Elegeram-se títulos originais, sem restrição de idioma e período e que abordassem gestantes com diagnóstico clínico e/ou laboratorial de COVID-19. Excluíram-se revisões, editoriais, títulos duplicados. As escalas de Newcastle-Ottawa (NOS, na sigla em inglês) e a de Murad et al. foram utilizadas para avaliar a qualidade dos estudos.

**Resultados** Foram incluídos 34 artigos com 412 gestantes infectadas pela síndrome respiratória aguda grave (SARS-Cov, na sigla em inglês) com idade média de 27,5 anos e média de 36,0 semanas gestacionais. O sintoma mais incidente foi a febre (49,7%;205). e 89 (21,6%) gestantes evoluíram para pneumonia viral grave. Os exames laboratoriais demonstraram aumento da proteína C reativa (37,8%; 154) e os radiológicos mostraram pneumonia com padrão em vidro fosco periférico (51,4%; 172). O parto cesáreo de emergência foi indicado para a maior parte das gestantes, e a complicação gestacional mais comum foi a ruptura prematura de membranas ovulares (3,4%; 14). Foram detectados 2 (0,5%) mortes neonatais, 2 (0,5%) natimortos, e 1 (0,2%) morte materna.

**Conclusão** Gestantes com doença coronavírus (COVID-19, na sigla em inglês apresentaram quadro clínico semelhante a gestantes não infectadas, com poucas repercussões obstétricas ou neonatais. Houve uma maior indicação de partos cesáreos antes do agravamento da doença e não se observaram evidências de transmissão vertical da infecção.

## Introduction

At the end of December 2019, in Wuhan, capital of the Hubei province, located in China, there was an outbreak by a virus that suddenly worried the authorities, due to their lack of knowledge and the rapid spread of the virus to other countries. The virus, belonging to the coronavirus family, was named severe acute respiratory syndrome (SARS-CoV2), and the illness caused by it was called coronavirus disease 2019 (COVID-19). This virus causes a highly transmissible infectious disease that can entail mild symptoms, such as dry cough, fever, odynophagia, or even severe acute respiratory syndrome. 1 2

Given the accelerated spread of the virus among countries, in March 2020, the World health Organization (WHO) declared a status of pandemic, and the beginning of the need for measures of social distance, with the purpose of avoiding the crowding of people and containing the spread of such infection. Data for the month of June 2020 already highlight more than 10 million confirmed COVID-19 cases worldwide and ∼ 500 thousand deaths. 3 4

Nevertheless, based on the information referring to the other aforementioned coronaviruses, SARS-CoV and middle east respiratory syndrome coronavirus (MERS-CoV), we noted a higher incidence of abortions, growth restriction, preterm births, and fetal death. In addition, these viruses in pregnant women determined a high number of complications, such as hospitalization in intensive care units (ICU), the need for assisted ventilation, renal failure, and death. 3

These complications are erroneously explained by the understanding of pregnancy as a condition of immunosuppression, which is a misconception, since pregnancy represents a peculiar immune condition that is modulated, but not suppressed. The correct concept allows caregivers and policy makers to make valid recommendations for the treatment of pregnant women during pandemics. Accordingly, in this COVID-19 pandemic, health care professionals need to understand the spectrum of presentations and outcomes of COVID-19 infection during pregnancy and childbirth. 5

Systematic reviews 5 6 7 8 published recently demonstrated good obstetric results for pregnant women with COVID-19; however, there are still gaps on the influence of infection on the choice of life of delivery, fetal and neonatal repercussions, as well as the possibility of vertical transmission. This study, therefore, aims to contemplate these points still little discussed and strengthen the knowledge about the behavior of the virus during pregnancy and perinatal. Furthermore, it aims to concentrate the best evidence, since most of the published works are case reports/series, in Chinese, which makes it difficult to homogenize information and summarize perinatal results in pregnant women with COVID-19.

Thus, a systematic literature review was performed to analyze the clinical and obstetric aspects of COVID-19 in pregnant women.

## Methods

This is a systematic literature review developed in accordance with the Preferred Reporting Items for Systematic Reviews and Meta-Analyses (PRISMA) recommendation. 9 The study protocol and review were not registered with PROSPERO due to the need for urgent information. We sought to answer research questions: “What are the clinical and obstetric repercussions in pregnant women infected with SARS-Cov-2?”

The search strategy was held in the following electronic bibliographic databases: Medical Literature and Retrieval System online (MEDLINE / PubMed); Latin American and Caribbean Literature on Health Sciences (LILACS); Scientific Electronic Library Online (SCIELO); Chinese National Knowledge Infrastructure (CNKI). In addition, we considered secondary research and gray bases in the pertinent literature in other sources, such as Google Scholar and OPENGREY. The reference section of the included studies was manually searched for additional relevant studies.

The search strategy included only key terms, according to a preestablished acronym of PICO (Population/Intervention/Comparison/Outcome). The search strategy that combines medical subject headings (MeSH) terms and free text words that will be used in MEDLINE (PubMed) and adjusted to other electronic databases, in the following manner: *Pregnant Women* AND ( *coronavirus* OR *coronavirus* OR *COVID-19* OR *2019-nCoV* OR *SARS-CoV-2* ) AND *Pregnancy* . The strategy details are presented in Table 1 .

**Table 1:** Search strategy, according to PICO

PICO	MEANING	DESCRIPTORS
**Population (P):**	Pregnant women of any age	((“Pregnant Women” [MeSH Terms]))
**Intervention (I):**	COVID-19 infection	((“coronavirus” [MeSH Terms] OR “coronavirus” [All Fields]) OR (“COVID-19” [All Fields] OR “2019-nCoV” [All Fields] OR “SARS-CoV-2” [All Fields]).
**Comparison (C):**	Non-pregnant women	Not applicable
**Outcomes (O):**	COVID-19 interference in the pregnancy	((“Pregnancy” [MeSH Terms]))

The eligibility criteria included complete original primary studies, available online in the selected databases and published in any languages, with no time frame, that addressed pregnant women with a clinical and/or laboratory diagnosis of COVID-19. All primary designs were considered, including case reports and case series. Given the infancy of the pandemic, urgent need of guidance, and limited higher quality information available on the topic, this was deemed reasonable. The exclusion criteria were literature reviews, editorials, or studies in which it was not possible to identify a relationship with the theme and duplicates in the databases as well as unreported maternal or perinatal results.

In the databases, we defined a peer search with specific descriptors, from March to April 2020. The search for data ended on May 13 ^th^ , 2020. The selection was made in pairs, with three authors, who independently selected the titles of the articles and then proceeded to read all abstracts. Those who complied with the study object were read in full. We considered original articles whose theme was related to pregnant women with COVID-19, regardless of the employed diagnostic technique. After selecting the articles, we held consensus meetings to confirm the equality of the selected articles. If there was disagreement, a fourth author was invited to give his/her opinion.

After agreement with the selected studies, we used a form developed by the authors to manually extract the following data:

- Variables related to the studies: authorship, year, country, method, population, and sample;
- Maternal variables: age, gestational age, signs, and symptoms of the pregnant woman with COVID-19, radiological and laboratory manifestations, gestational repercussions (delivery, obstetric complications), complications and clinical outcomes (respiratory failure, maternal mortality);
- Perinatal variables: perinatal complications (fetal distress, low birth weight, prematurity), vertical transmission of COVID-19, perinatal mortality.

The primary outcomes were to identify obstetric repercussions, such as delivery route, gestational complications, severity of SARS-Cov-2 infection, and mortality; the fetal repercussions were complications such as fetal distress, fetal and neonatal mortality, and vertical transmission of COVID-19. The severity was assessed based on pregnant women who developed severe pneumonia or severe acute respiratory syndrome (SARS), requiring oxygen supply.

The quality of the studies was based on checking international guidelines. For case studies, we used the tool proposed by Murad et al. (2018), 10 composed of four domains: selection, verification, causality, and communication. According to the recommendation, we made a general judgment about the methodological quality based on the questions considered more critical in the specific clinical setting. The quality was considered either satisfactory or unsatisfactory, with satisfactory being when at least 1 item from each domain of the instrument was met. We used the Newcastle-Ottawa Scale (NOS) 11 for cohort studies in the cases of observational studies. The NOS uses a star system (0–9) to assess the selected studies in 3 domains: selection (0–4), comparability (0–2), and results (0–3). Higher scores represent better quality. and then, we considered: strong evidence (6–9 points), moderate evidence (4–5 points), and limited evidence (< 4 points).

The studies were presented and synthesized in tables with exposure of the most relevant data, and the treatment of these tables was performed in the Microsoft Office Excel software (Microsoft Corp., Redmond, WA, USA), to determine the percentage of incidence of the variables.

## Results

**Fig. 1:**
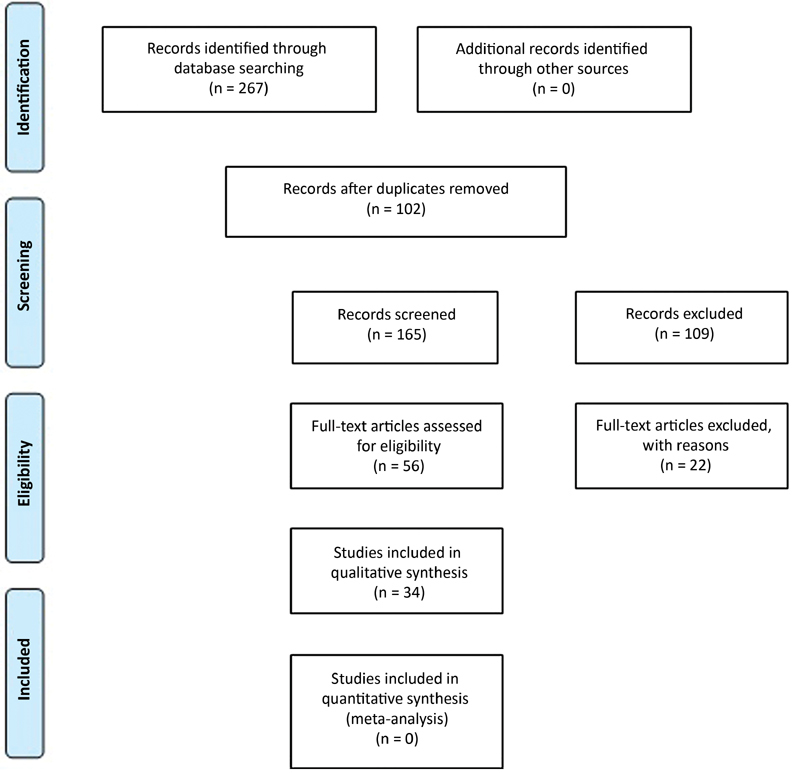
Flowchart of PRISMA selection.

From the total of 267 identified articles, 102 were eliminated because they were duplicated in the databases. One hundred and sixty-five titles and abstracts were read, and, of these, 56 manuscripts were chosen to be read in full. After eliminating those that did not meet the object of the study, 34 studies were selected for analysis, according to the flowchart ( Fig. 1 ).

As for the method, most studies were 1 or multiple case reports (23; 67.6%) and observational (11; 32,4%), with clinical and epidemiological characterization of pregnant women with COVID-19. The quality of the studies is displayed in supplementary tables S1 and S2 . In supplementary table S1 , we represented the quality of the studies of one or multiple cases, according to the tool of Murad et al. (2018), 10 in which we observed a satisfactory quality in all studies. Table 2 shows the quality of the studies, according to the NOS scale. Quality scores ranged from 6 to 9 points, which denotes studies with strong evidence ( Supplementary tables S1 and S2 ).

**Table 2:** Characterization of the selected studies on COVID-19 in pregnant women. Brazil, 2020

Art	Author/ year/ country;	N	Diagnostic method	Maternal age	Trimester average GA	Main signs and symptoms	Radiological/laboratory manifestations	Maternal/gestational/fetal repercussions
**1**	Xiong et al. (2020) 12 ; China	1	RT-PCR	(25)	2 ^nd^ (26 GW)	38°C fever, dry cough, and tremor	Radiological: pulmonary opacity Laboratory: neutrophilia and lymphocytopenia	Without repercussions
**2**	Li et al. (2020) 15 ; China	16	RT-PCR	(31,5)26–37	3 ^rd^ (35 GW)	Fever (4;25.0%)	Radiological: bilateral pneumonia 43.75% (7) and unilateral pneumonia 50% (8) Laboratory: leukopenia and neutrophilia	14 (87.5%) Cesarean deliveries 3 (18.75%) Premature births, of which: 2 (66.67%) premature rupture of ovarian membranes (RPMO) and 1 (33.3%) bleeding 2 (12.5%) fetal distress
**3**	Chen et al. (2020) 27 ; China	5	RT-PCR	(28) 25–31	3 ^rd^ (39 GW)	Fever (after delivery) and dry cough (2;40.0%)	Radiological: bilateral pneumonia 60% (3) and 40% (2) unilateral Laboratory: increased CRP (4; 80.0%) and alkaline phosphates (4; 80.0%); leukopenia 40% (2)	2 (40.0%) Cesarean deliveries
**4**	Yu et al. (2020) 13 ; China	7	RT-PCR	(32) 29–34	3 ^rd^ (39 GW)	Fever (86%;6), cough, dyspnea, and diarrhea (14%;1, each)	Radiological: 86% (6) bilateral pneumonia and 14% (1) unilateral Laboratory: neutrophilia (71%; 5), lymphopenia (71%; 5), thrombocytopenia (29%; 2) and elevation of D-dimer (100%; 7), alanine aminotransferase (29%; 2) and CRP (100%; 7)	Infected NB 36h after delivery
**5**	Kang et al. (2020) 16 ; China	1	RT-PCR	30	3 ^rd^ (35 GW).	Cough	Radiological: pneumonia and bilateral pleural effusion Laboratory: Without changes	Emergency cesarean delivery Fetal bradycardia
**6**	Liu et al. (2020) 45 ; China	15	RT-PCR	32 (23–40)	1 ^st^ to 3 ^rd^ (12 to 38 GW).	Fever (86.6%; 13); Cough (60.0%; 9); fatigue (26.7%; 4); myalgia (20.0%; 3); dyspnea, odynophagia, and diarrhea (6.6%; 1, each)	Radiological: ground-glass opacity and late pulmonary consolidations Laboratory: lymphocytopenia (80.0%; 12) and increased value of C-reactive protein (66.6%; 10)	10 (66.6%) cesarean deliveries 3 (20.0%) premature births
**7**	Chen et al. (2020) 14 ; China	9	RT-PCR	30 (26–40)	3 ^rd^ (37 GW)	Fever (77.7%; 7); Cough (44.4%; 4); myalgia (33.3%; 3); odynophagia and malaise (22.2%; 2, each)	Radiological: ground-glass opacity (88.8%; 8) Laboratory: lymphocytopenia (55.5%; 5), elevation of aminotransferases (33.3%; 3) and C-reactive protein-PCR (66.6%; 6)	9 (100.0%) cesarean deliveries 4 (44.4%) newborns had birth weight less than 2500 g 1 (11.1%) PROM
**8**	Liu et al. (2020) 17 ; China	13	RT-PCR	29 (22–36)	2 patients in the 2 ^nd^ (26 GW); 11 patients in the 3 ^rd^ (35 GW)	Fever and fatigue (10; 77%); Dyspnea (3; 23%)	NR	10 (77%) cesarean deliveries, 5 (38.4%) of which were emergency 6 (46%) premature birth 3 (23%) fetal distress. 1 (7.6%) PROM 1 (7.6%) stillborn 1 (7.6%) maternal multiple organ dysfunction syndrome (MODS)
**9**	Li et al. (2020) 18 ; China	1	RT-PCR	30	3 ^rd^ (35 GW)	Dry cough, fever, and dyspnea	Radiological: bilateral irregular pulmonary infiltrates Laboratory: slightly abnormal.	Emergency cesarean delivery Fetal bradycardia
**10**	Chen et al. (2020) 19 ; China	3	RT-PCR	29,6 (23–34)	3 ^rd^ (36.6 GW)	Pre or postnatal fever (100%, 3) and chest pain (33.3%; 1).	Radiological: bilateral ground-glass opacities and bronchiectasis (100%, 3) Laboratory: significant increase in CRP (100%, 3)	3 (100.0%) cesarean deliveries 1 (33.3%) premature birth 1 (33.3%) premature placental displacement 1 (33.3%) placenta previa 1 (33.3%) NB with LBW
**11**	Zhang et al. (2020) 20 ; China	16	RT-PCR	29 (24–34)	3 ^rd^ (38 GW)	Cough; Chest pain; dyspnea, and diarrhea	NR	16 (100%) emergency cesarean section 3 (18.7%) premature births 2 (12.5%) fetal distress 3 (18.7%) rupture of membranes 1 (6.25%) fetal distress and fetal bradycardia 1 (6.25%) severe maternal pneumonia
**12**	Yan et al. (2020) 21 ; China	116	RT-PCR	31 (24–41)	3 ^rd^ (38 GW) 1 ^st^ (4;3.4%) 2 ^nd^ (6;5.2%) 3 ^rd^ (96;91.4%)	27 (23.3%) asymptomatic Fever (50.9%; 59), cough (28.4%; 33), fatigue (12.9%; 15), dyspnea (7.8%; 9); odynophagia (8.6%; 10); myalgia (5.3%; 6); diarrhea (0.9%; 1)	Radiological: bilateral ground-glass pneumonia (96.3%; 104) Laboratory: lymphopenia and increased CRP (44.0%; 51), leukopenia (24.1%; 28)	99 deliveries, 85 (85.9%) of which were cesarean and 33 (38.8) indications due to COVID-19 21 (21.2%) premature births 6 (6.0%) PROM 1 abortion 1 neonatal death from asphyxiation. 8 (6.9%) severe pneumonia with ventilatory support
**13**	Ferrazzi et al. (2020) 22 ; Itália	42	RT-PCR	NR	NR	Dyspnea (20; 48%)	Radiological: interstitial pneumonia (20; 48.0%) Laboratory: NR	18 (43%) cesarean deliveries 2 (4.7%) premature births 7 (35%) severe pneumonia with ventilatory support
**14**	Kalafat et al. (2020) 23 ; Turquia	1	RT-PCR	32	3 ^rd^ (35 GW and 3 days)	Cough (1; 100%), dyspnea (1; 100%)	Radiological: bilaterally thick pulmonary B lines, located in the posterior basal segments of the lung Laboratory: NR	Premature cesarean delivery Reduced fetal movements
**15**	Wen et al. (2020) 24 ; China	1	RT-PCR	31	3 ^rd^ (30 GW)	Diarrhea. (1; 100%)	Radiological: irregular consolidation and bilateral ground-glass opacities, mainly distributed in the sub-plastic regions. Left pleural effusion Laboratory: NR	Without gestational changes
**16**	Fan et al. (2020) 25 ; China	2	RT-PCR	31 (29–34)	3 ^rd^ (36.5 GW)	Chills, fever (37.6 ± 38.5°C), nasal congestion and odynophagia	Radiological: irregular consolidation and bilateral opacities Laboratory: lymphopenia (1; 50.0%)	2 (100%) Emergency cesarean delivery 2 (100%) NB had mild neonatal pneumonia
**17**	Pierce-Williams et al. (2020) 26 ; EUA	64	RT-PCR	33	3° (29.9 GW)	Fever, dyspnea	Radiological: NR Laboratory: elevation of ferritin, liver enzymes, CRP, interleukin-6, d-dimer, LDH, creatine phosphokinase (CPK)	44 (69.0%) severe pneumonia 20 (31.0%) critical illness 14 (22.0%) SARS 24 cesarean deliveries 31 (48.4%) premature births, 15 in critical patients 19 (30.0%) invasive mechanical ventilation 1 cardiorespiratory arrest, without maternal mortality 3 (9.0%) postpartum hemorrhage 1 neonatal COVID-19 infection
**18**	Blauvelt et al. (2020) 28 ; EUA	01	RT-PCR	34	3 ^rd^ (28 GW)	Asthenia, fever, myalgia, cough, and dyspnea	Radiological: bilateral opacities in the lower lobes Laboratory: lymphopenia and lactate elevation, D-dimer, and CRP	Cesarean and premature delivery SARS, with the need to undergo invasive mechanical ventilation NB with respiratory distress
**19**	Hong et al. (2020) 29 ; China	01	RT-PCR e laboratorial	36	2 ^nd^ (23 weeks)	Fever, myalgia and dyspnea, cough, tachycardia	Radiological: opacities in pulmonary bases. Laboratory: lymphopenia and elevation of transaminases, CPK, and CRP	SARS, with the need to undergo invasive mechanical ventilation
**20**	Li et al. (2020) 30 ; China	01	RT-PCR	31	3 ^rd^ (35 GW)	Odynophagia, dry cough, fever, and dyspnea	Radiological: diffuse opacity in bilateral ground glass Laboratory: lymphopenia, respiratory acidosis and abnormal coagulogram, elevated CRP and interleukin 6	Premature cesarean delivery SARS with multiple organ dysfunction Neonatal death
**21**	Schnettler et al. (2020) 31 ; EUA	01	RT-PCR	39	3 ^rd^ (31 GW)	Cough, dyspnea, fever, and asthenia	Radiological: peripheral diffuse opacities in bilateral ground glass Laboratory: leukopenia. lymphopenia, thrombocytopenia, elevated transaminases	Emergency and premature cesarean delivery. SARS, with the need to undergo invasive mechanical ventilation
**22**	Wu et al. (2020) 32 ; China	08	RT-PCR	29 (26–35)	3 ^rd^ (38 GW)	Fever on admission (1;12.5%), and fever in the postpartum period (3; 37.5%)	Radiological: peripheral diffuse opacities in bilateral ground glass (6; 75.0%) Laboratory: leukocytosis (6; 75.0%); lymphopenia (5; 62.5%), elevation of D-dimers and creatine kinase (CK) and creatine kinase-MB (CK-MB) in 4 (50.0%) in the postpartum period	6 (75.0%) Cesarean delivery 1 fetal distress
**23**	Peng et al. (2020) 33 ; China	01	RT-PCR	25	3 ^rd^ (35 SG)	Fever, fatigue and dyspnea	Radiological: peripheral diffuse opacities in bilateral ground-glass pattern Laboratory: NR	Emergency cesarean and premature delivery Fetal distress
**24**	Breslin et al. (2020) 34 ; EUA	43	RT-PCR	27 (20–39)	3 ^rd^ (37 SG)	Cough (19; 65.6%); fever (14; 48.3%); myalgia (11; 37.9%). headache (8; 27.6%); dyspnea (7; 24.1%); chest pain (5; 17.2%)	Radiological: peripheral diffuse opacities in bilateral ground-glass pattern (1; 2.32%) Laboratory: NR	Cesarean delivery (8; 44.4%) Premature birth (4; 13.8%) 1 PROM 2 (4.6%) severe maternal pneumonia 1 newborn with respiratory distress
**25**	Karami et al. (2020) 35 ; Irã	01	RT-PCR	27	3 ^rd^ (30 SG)	Dyspnea, fever, cough, myalgia, and tachypnea	Radiological: bilateral diffuse peripheral ground-glass opacities and pleural effusion Laboratory: leukopenia, thrombocytopenia, elevated CRP, lactic dehydrogenase (LDH) Echocardiogram: severe systolic dysfunction right	Maternal death from severe acute respiratory syndrome (SARS) and MODS; stillborn
**26**	Iqba et al. (2020) 36 ; EUA	01	RT-PCR	34	3 ^rd^ (39 GW)	Fever, cough, chills, and myalgia	Radiological: diffuse pulmonary opacities Laboratory: lymphopenia	NR
**27**	Xia et al. (2020) 37 ; China	01	RT-PCR	27	3 ^rd^ (36 GW)	Fever, cough, drop in oxygen saturation (92%)	Radiological: bilateral diffuse peripheral ground-glass opacities Laboratory: neutrophilia, lymphopenia, and elevated CRP	Cesarean delivery Fetal distress
**28**	Buonsenso et al. (2020) 38 ; Itália	07	RT-PCR	40	3 ^rd^ (36 GW)	Cough	Radiological: diffuse interstitial lung disease Laboratory: NR	2 (28.5%) Cesarean delivery 1 Premature Birth 1 Abortion 2 Neonatal COVID-19 infections
**29**	Qiancheng et al. (2020) 39 ; China	28	RT-PCR	30 (18–41)	3 ^rd^ (38 GW)	Fever (5; 17.9%), asthenia (1; 3.6%), cough (7; 25%), dyspnea (2; 7.1%), abdominal pain (5; 17.9%)	Radiological: bilateral peripheral diffuse ground-glass opacities (26; 92.9%) Laboratory: Leukocytosis (10; 35.7%), increased CRP (17; 68.0%), lymphopenia (8; 28.6)	17 (60.0%) Cesarean delivery 1 (4.3%) Premature birth 4 (14.2%) Induced abortion 2 (7.1%) Severe pneumonia
**30**	Lyra et al. (2020) 40 ; Portugal	01	RT-PCR	35	3 ^rd^ (39 GW)	Cough	NR	Cesarean delivery
**31**	Kelly et al. (2020) 41 ; EUA	01	RT-PCR	−	3 ^rd^ (33 GW)	Vomiting, cough, and tachycardia	Radiological: Radiography showed subsegmental atelectasis without consolidation Laboratory: lymphopenia and mild elevation of liver enzymes	Cesarean and premature delivery 1 SARS, with the need to undergo invasive mechanical ventilation
**32**	Browne et al. (2020) 42 ; EUA	01	RT-PCR e Serological (IgG/IgM)	33	2 ^nd^ (23 GW)	Fever, cough, and myalgia	Radiological: NR Laboratory: leukocytosis	Premature cesarean delivery
**33**	Indraccolo (2020) 43 ; Itália	01	RT-PCR	NR	3 ^rd^ (32 GW)	Cough, epigastric pain, and chest pain	NR	NR
**34**	Lu et al. (2020) 44 ; China	01	RT-PCR	22	3 ^rd^ (38 GW)	Asymptomatic	Radiological: bilateral pleural effusion Laboratory: hyperproteinemia, elevated transaminases and LDH	Cesarean delivery
**Total**	**N** 412	**Diagnostic method** RTC-PCR (412;100%)	**Average age** 27.5		**GA** 36.0	**Symptoms** Fever (49.7%; 205); dyspnea (31.5%; 130); cough (26.5%; 109), fatigue (8.2%; 34); myalgia (7.0%; 29); chest pain (5.5%; 23), diarrhea (4.8%; 20); odynophagia (3.6%; 15)	**Main radiological findings** Pneumonia/ground-glass pattern (51.4%; 212), 51.5% (172) of which were bilateral **Main laboratory findings** Increased CRP (37.8%; 154); lymphopenia (20.3%; 84); leukopenia (14.2%; 58); neutrophilia (5.5%; 23);	**Maternal and Gestacional repercussions** Cesarean delivery (36.2%; 149); premature birth (18.4%; 76); abortion (1.0%; 4); PROM (3.4%; 14); hemorrhage (1.0%; 4); placental abruption (0.2%; 1);p previa (0.2%; 1); severe maternal pneumonia (21.6%; 89); maternal death (0.2%; 1); **Fetal repercussions** Stillborn (0.5%; 2); neonatal death (0.5%; 2); fetal distress (2.7%; 11); fetal bradycardia (0.7%; 3); LBW (1.0%; 4); neonatal COVID infection (1.0%; 4)

Abbreviations: CRP, C-reactive protein; GA, gestational age; GW, gestational week; LBW, low birth weight; NB, newborn; NR, not reported; PROM, premature rupture of ovarian membranes; RT-PCR, reverse transcription polymerase chain reaction; SARS, severe acute respiratory syndrome.

## Description of the Study Findings

To compose the systematic review, we included 34 articles, all published in 2020, mainly produced in China (61.7%; 21) available in the PubMed/MEDLINE databases ( Table 2 ).

Altogether, 412 infected pregnant women, with an average age of 27.5 years and who were mostly in the 3 ^rd^ trimester of pregnancy, with an average of 36.0 gestational weeks, were assessed in the studies. All pregnant women were diagnosed with COVID-19 through the reverse transcription polymerase chain reaction (RT-PCR) method. The most frequent signs and symptoms were fever (205 [49.7%]) dyspnea (130 [31.5%]), cough (109 [26.5%]), fatigue (34 [8.2%]), myalgia (29 [7.0%]), chest pain (23 [5.5%]), diarrhea (20 [4.8%]) and odynophagia (15 [3.6%]) ( Table 2 ).

Among the most common findings in laboratory tests, we found elevated C-reactive protein (CRP) (154 [37.8%]), lymphopenia (84 [20.3%]), leukopenia (58 [14.2%]) and neutrophilia (23 [5.5%]). In the assessment by computerized tomography (CT) or chest X-ray, 51.4% (212) of the pregnant women presented a characteristic image of viral pneumonia with ground-glass pattern, with 172 (51.5%) having bilateral pulmonary involvement ( Table 2 ).

Among the main gestational complications, we found premature birth (76 [18.4%]), abortion (4 [1.0%]), and premature rupture of ovarian membranes (PROM) (14 [3.4%]). Severe maternal pneumonia took place in 89 (21.6%) of the pregnant women, with 1 (0.2%) maternal death being recorded. With regard to fetal repercussions, we detected 2 (0.5%) stillbirths, 2 neonatal deaths (0.5%), 11 cases of fetal distress (2.7%), 3 cases of fetal bradycardia (0.7%). And 4 cases of low birth weight (LBW) (1.0%), and 4 cases of neonatal COVID infection (1.0%) ( Table 2 ).

## Discussion

Coronaviruses are a large class of viruses, among which the newly discovered SARS-Cov-2 is the 7 ^th^ coronavirus currently known due to its capacity of infecting human beings. The disease caused by it, COVID-19, has become a challenging threat to public health worldwide; and when it comes to pregnant women, data on the outcomes of the disease are still limited. 12

We analyzed 34 studies involving 412 infected pregnant women. These papers were all from the current year, and there was a predominance of Chinese productions, probably because it was the region initially most affected by the disease and that, for this reason, gathered early evidence about the affected patients. The findings mainly highlighted young women who were in the third trimester of pregnancy. This is a relevant fact, since the results of the present study cannot be extrapolated to pregnant women with virus infection in the first or second trimester of pregnancy. 13 14

Pregnant women presented a clinical course similar to non-pregnant adult women. 12 14 15 16 17 18 19 20 21 22 23 24 25 26 The current data on COVID-19 infection suggests that pregnant women experience similar or even lower rates of serious illness compared with non-pregnant women; however, additional data are needed, and it is crucial to determine the peculiar factors in pregnant women who predict a more severe course of this disease, with to the goal of guiding the clinical management, as well as the ideal time of delivery. 26

The most frequent signs and symptoms were fever, dyspnea, cough, and fatigue, respectively. 8 9 10 We should underline that asymptomatic pregnant women were detected in the predelivery period and that they presented symptoms such as fever after delivery. 19 20 21 22 23 24 25 26 27 28 29 30 31 32 A total of 89 pregnant women evolved with severe viral pneumonia and/or SARS, and 24 required invasive ventilatory support. 17 26 28 29 30 31 32 33 34 35 36 37 38 39 40 41 We noted clinical pictures of multiple organ dysfunction and reversed cardiorespiratory arrest; 26 however, there was only one report of maternal death related to COVID-19. 35 The data indicate that 95% of the women who need intubation will do so 20 days after the onset of symptoms. 26

We can notice that most respiratory symptoms found in the studies were mild; however, pregnant women with comorbidities, such as preeclampsia, can have worse outcomes, since pneumonia can aggravate pulmonary edema and reduce oxygen saturation. 11 28 This is because pregnant women have a higher oxygen consumption, with an increase of 10 to 20%, changes in hormonal levels and decreased lung volumes, caused by an increase in the size of the uterus during pregnancy, as well as edema of the respiratory tract mucosa, which can cause a faster clinical deterioration and make pregnant women more susceptible to respiratory pathogens and severe pneumonia, because they are in a state of adaptive immunity. 13 14 42

Nevertheless, we should consider the high number of asymptomatic pregnant women in all studies and/or with atypical symptoms, which emphasizes the need to improve the investigation of COVID-19 upon admission, as well as the strengthening of infection control measures. 15 21 43 44

Among the most common laboratory changes, we found increased CRP, lymphopenia, and leukopenia. It is recommended that, in addition to the RT-PCR test as the gold standard for the diagnosis of COVID-19-related pneumonia, laboratory tests and a comprehensive assessment of the medical history of the patient, epidemiological exposure and symptoms are accomplished. 14

The radiological findings, mainly by chest CT, demonstrated pulmonary opacities with peripheral ground-glass pattern with bilateral pulmonary involvement, suggestive of viral pneumonia. In most cases, the tomographic images obtained before and after delivery did not show signs of aggravated pneumonia, indicating that pregnancy and childbirth do not seem to aggravate the course of the tomographic characteristics. 45 Given the difficulty and delay in RT-PCR tests, low-dose chest CT, or even the use of chest ultrasound, 23 may be an effective method to screen for COVID-19-related pneumonia in pregnant women in the third trimester. 15 In critical areas, radiological findings consistent with COVID-19 infection may emerge before the results of RT-PCR. 23

Regarding the gestational repercussions, we noted a greater indication of emergency cesarean deliveries for infected pregnant women, many of these involving preterm infants. It is important to emphasize that the uncertainty about the risk of vertical transmission through vaginal delivery or early delivery before the disease aggravation were the main reasons for the recommendation of cesarean sections. 14 In these cases of emergency cesarean sections, maternal clinic should be assessed, giving preference to spinal anesthesia, to reduce the impact on the respiratory circulation of the mother and her baby. If the infected pregnant woman is critical, general anesthesia is recommended for tracheal intubation. 16 37

Management of expectant support of the pregnant woman is reasonable for most 42 ; however, for women over 34 gestational weeks and with associated risk factors, cesarean delivery can be indicated early before the onset of the critical illness. 26 As for the event of severe maternal infection, delivery should be postponed until pulmonary stability is achieved, unless the pregnancy has reached its term. 31 A study demonstrated that pregnant women with COVID-19 presented a higher factor indicating cesarean delivery due to muscle fatigue and uterine contraction (1.3 ± 0.6) than the control group without infection [(0.5 ± 0.7)] ( *p* -value = 0.001). Pregnant women with COVID-19 are in an infected and hypoxic state with greater inflammatory factors, increased uterine contractility and a higher risk of postpartum hemorrhage. The indication of termination of pregnancy depends on the status of the illness, but terminating it early may improve maternal lung function. 26

The studies were not in agreement regarding the relationship between COVID-19 and preterm births. A retrospective study of data analysis of 116 pregnant women did not find association among the gestational infection by SARS-Cov-2, the increase in premature births, and the risk of abortion. 21 Nevertheless, other studies corroborate that there is a higher incidence of premature births in confirmed cases of COVID-19, when compared with non-infected pregnant women, 15 42 and that the need for the accomplishment of this procedure is greater in women with critical infection, to whom the indication is given, mainly, by maternal status, and not fetal. 26 28

The most common gestational complication was the PROM 15 17 ; however, in the studies, it was not possible to clarify the direct relationship between this complication and the SARS-Cov-2 infection. Nevertheless, it is known that inflammatory reactions caused by infection by the virus can impair the development and the function of the placenta, especially in blood vessels, resulting in adverse outcomes in pregnancy. 19

Regarding fetal manifestations, deliveries resulting in two stillborn 8 14 and two neonatal deaths 21 30 were detected, as well as cases of respiratory distress, 28 fetal distress, 15 17 20 32 37 with fetal bradycardia 16 19 20 and LBW. 14 19 Severe SARS-Cov-2 infection can change the fetal intrauterine environment, and the inflammatory storm caused by the infection triggers a systemic immune response that can also attack fetal organs. 30 Despite this frequency of complications, most studies have shown that NBs from infected mothers do not present significant differences in the main indicators of neonatal complications when compared with NBs from non-infected mothers. 15

We did not find occurrence of vertical transmission of COVID-19. 19 25 33 38 39 44 Tests with an oropharynx sample from 2 NBs were positive with 36 hours and 48 hours after birth, 13 26 and 2 other NBs were positive two weeks later. 38 Surveys investigating SARS-CoV-2 in maternal serum, placenta, umbilical cord, amniotic fluid, vaginal swabs, and breast milk were negative in the nucleic acid test in all studies that cited them, which precludes vertical intrauterine transmission. Premature neonates may be at particular risk of perinatal COVID-19 infection, since the active transfer of protective maternal immunoglobulins does not reach its peak before 28 to 30 weeks of life, besides the immature integrity of the skin of NBs, which may be a risk factor for perinatal transmission. 28

With regard to breastfeeding, as it is a recent infection, there is currently insufficient evidence to support the presence of SARS-CoV-2 in breast milk; however, breastfeeding by infected mothers, in part of the studies, is not recommended, according to the experience related to SARS. 20 25 27 Nevertheless, an Italian consensus eases this indication by admitting that COVID-19-positive mothers with mild or asymptomatic symptoms can breastfeed while wearing a surgical mask. 22 And those infected and symptomatic should be separated from their NBs, but they can perform weaning to provide their children with breast milk. 34

Four systematic reviews currently published on COVID-19 and pregnant women 5 6 7 8 were composed of 9, 6, 24, and 33 studies, the majority of which were case series. Until the moment of completing this search in databases, this systematic review is the one that addressed the largest number of studies and the largest sample of pregnant women infected with SARS-Cov-2.

The present systematic review demonstrated a low maternal mortality rate, as well as a reduced incidence of complications, such as SARS, concluding that COVID-19-positive pregnant women have fewer symptoms than the general population and that this infection is not associated with poor perinatal results. 5 6 7 8 One concern, however, was related to the high rate of premature delivery by cesarean section in these women. 6

This review considers the results of the aforementioned research when concluding that the maternal, fetal, and neonatal outcomes in COVID-19 were, in most studies, positive. And it brings new knowledge to the fact that pregnant women can remain asymptomatic or when they have symptoms, fever is their main one, accompanied by the elevated serum CRP and that cesarean delivery is a recurrent outcome in this type of infection. The absence of vertical transmission was reinforced; however, neonatal COVID-19 infection was demonstrated, which serves to reinforce the need for infection control measures at the time of delivery.

Nevertheless, we should underline that the average age of 27.5 years of women in this study reveals that, as in the general epidemiological panorama of the disease, young people are less prone to COVID-19 complications and this factor may be responsible for the reduced number of negative outcomes, which can, consequently, undermine a possible protective factor provided by pregnancy.

We should also underline that the findings of this systematic review predominantly bring characteristics of eastern, Chinese, and North American women, which prevents further generalization of the data, due to phenotypic, cultural, and structural differences in health services. Accordingly, more studies are needed to assess the profile of pregnant women from other countries with a high incidence of the disease, such as Brazil, with a view to comparing the findings.

The present review has limitations, because, as a result of the COVID-19 pandemic, new studies are published daily, and it listed works until May 13 ^th^ , 2020. Moreover, the analyzed studies, since they are mostly with case study methods, add up to a small sample of pregnant women and, in addition, despite presenting satisfactory quality, according to the assessment tools, they consist of studies with low evidence. Nevertheless, we believe that the tendency toward the manifestation of symptoms in pregnant women infected with SARS-CoV-2 does not differ much from what was exposed in the results.

Furthermore, the findings of the current study characterized women in the third trimester of pregnancy, and it is not possible to generalize such considerations to pregnant women in the first and second trimesters. Therefore, there is a need for studies that focus on this viral infection in earlier gestational periods.

## Conclusion

The course of infection in pregnant women with SARS-Cov-2 is similar to the one in non-infected pregnant women. We did not find occurrence of vertical transmission of COVID-19. The main symptom presented by pregnant women with COVID-19 was fever; however, most of the patients were asymptomatic. The most common laboratory change was the increase in CRP, and the radiological findings were peripheral pulmonary opacities with ground-glass pattern. There was a greater indication of emergency cesarean deliveries for infected pregnant women, many of these involving preterm infants; however, studies have not shown consensus on the risk of prematurity associated with COVID-19. The main fetal manifestations were fetal distress, with reduced fetal heart rate, but not directly related to SARS-Cov-2. Mortality consisted of two stillborn, two neonatal deaths, and one maternal death.
