# Supplementary material for: Clinical and Obstetric Aspects of Pregnant Women with COVID-19: A Systematic Review
Source: Rev Bras Ginecol Obstet. 2021 Dec 21;43(12):949–60. doi: 10.1055/s-0041-1733913 (PMC10183856; doi:10.1055/s-0041-1733913)
Supplement: Supplementary file 1 — Supplementary Material [file 10-1055-s-0041-1733913-s200174.pdf]

Supplementary Table S1 Assessment of the quality of studies in one or multiple cases, according to the tool by Murad et al. (2018)[10]

Author, Year	Study Method	CS and MCS Quality	
		Satisfactory	Unsatisfactory
Xiong et al. (2020)[(12)]	CS	X	
Chen et al. (2020)[(14)]	MCS	X	
Yu et al. (2020)[(15)]	MCS	X	
Kang et al. (2020)[(16)]	CS	X	
Liu et al. (2020)[(17)]	MCS	X	
Li et al. (2020)[(20)]	CS	X	
Chen et al. (2020)[(21)]	MCS	X	
Kalafat et al. (2020)[(25)]	CS	X	
Wen et al. (2020)[(26)]	CS	X	
Fan et al. (2020)[(27)]	MCS	X	
Blauvelt et al. (2020)[(29)]	CS	X	
Hong et al. (2020)[(30)]	CS	X	
Li et al. (2020)[(31)]	CS	X	
Schnettler et al. (2020)[(32)]	CS	X	
Peng et al. (2020)[(34)]	CS	X	
Karami et al. (2020)[(36)]	CS	X	
Iqbal et al. (2020)[(37)]	CS	X	
Xia et al. (2020)[(38)]	CS	X	
Lyra et al. (2020)[(41)]	CS	X	
Kelly et al. (2020)[(42)]	CS	X	
Browne et al. (2020)[(43)]	CS	X	
Indraccolo (2020)[(44)]	CS	X	
Lu et al. (2020)[(45)]	CS	X	

Abbreviations: CS, case study; MCS, multiple case study.

Supplementary Table S2 Assessment of the quality of observational studies, according to the NOS scale

Author, Year	Study Method	OS Quality			Final Score
		Selection	Comparison	Result	
Li et al. (2020)[15]	OS	4 *	2 *	3*	9
Liu et al. (2020)[17]	OS	3*	2*	3*	8
Chen et al. (2020)[19]	OS	4 *	2 *	3*	9
Zhang et al. (2020)[20]	OS	4*	2*	3*	9
Yan et al. (2020)[21]	OS	4*	2*	3*	9
Ferrazzi et al. (2020)[22]	OS	4*	2*	3*	9
Pierce-Williams et al. (2020)[26]	OS	4*	2*	3*	9
Wu et al. (2020)[32]	OS	4*	2*	3*	9
Breslin et al. (2020)[34]	OS	4*	1*	3*	8
Buonsenso et al. (2020)[38]	OS	3*	1*	2*	6
Qiancheng et al. (2020)[39]	OS	4*	2*	3*	9

Abbreviation: OS, observational study.
Strong evidence (6–9 points), moderate evidence (4–5 points) and limited evidence (< 4 points).

 © 2021. Federação Brasileira de Ginecologia e Obstetrícia. All rights reserved.
